# Prevalence of COVID-19 in Different Types of Collagen Vascular Diseases and its Relationship with Drugs Used in these Patients

**DOI:** 10.31138/mjr.33.3.311

**Published:** 2022-09-30

**Authors:** Simin Almasi, Elahe Mirzazade, Dorsa Barzi, Babak Eshrati

**Affiliations:** 1. Department of Rheumatology, Firouzgar Hospital, Iran University of Medical Science, Tehran, Iran,; 2. Firoozabadi Clinical Research Development Unit (FACRDU), Iran University of Medical Science (IUMS), Tehran, Iran,; 3. Faculty of Veterinary Medicine, University of Tehran, Tehran, Iran,; 4. School of Medicine, Preventive Medicine and Public Health Research Center, Social Injury Prevention Research Institute, Iran University of Medical Science

**Keywords:** COVID-19, collagen vascular, disease

## Abstract

**Objectives:**

The outcome of COVID-19 disease in collagen vascular disease and its comparison with other infected people in the community, are not fully understood yet.In this study, we examined whether the prevalence and severity of COVID-19 in these patients is higher than the general population or not?

**Methods:**

This cross-sectional study was performed between August and December 2020 on collagen vascular patients referred to the rheumatology clinic of Firouzgar Hospital. Patients were evaluated for a history of COVID-19. The prevalence of the and its relationship with age, sex, type of disease, medications, blood. The history of influenza vaccine was also evaluated in these patients.

**Results:**

Among the total of 748 patients, 574 (76.6%) subjects were women, and 174 (23.3%) subjects were men. The mean age of the patients was 47.46 ± 13.56 years old. The prevalence of COVID-19 was 8.0% and its highest prevalence was related to rheumatoid arthritis (36.7%) and the lowest one was for vasculitis (1.7%). Notably, 12.5% of the patients who did not suffer from COVID-19, were vaccinated against influenza (p-value = 0.54). In this regard, a significant relationship was found between COVID-19 prevalence and previous existence of interstitial lung disease (p -value = 0.017).

**Conclusions:**

The prevalence of COVID-19 in collagen vascular patients was not higher than the general population. There was also no significant relationship between the prevalence of COVID-19 and its severity and collagen vascular patient’s blood group, the type of their disease and drug. More studies are required on the effect of DMARD drugs on the prevalence and severity of COVID-19 disease in collagen patients. Better results could be obtained if this study is done with a larger sample size.

## INTRODUCTION

Coronaviruses are a large family of viruses that can infect both animals and humans.

A large number of known corona viruses have caused many respiratory infections in humans ranged from a common cold to more serious illnesses such as Middle East Respiratory Syndrome (MERS) and Severe Acute Respiratory Syndrome (SARS).

In some of the studied patients, up-regulation of pro-inflammatory cells and cytokines consequently caused Cytokines Release Syndrome (CRS) and severe ARDS and symptoms similar to hemophagocytic lymphohistiocytosis (sHLH).^1^

COVID-19 and its associated disease remained unknown until the recent outbreak in Wuhan, China, in December 2019.^2^

One of the concerns with the global spread of the disease in rheumatic patients is that these patients are prone to bacterial and viral infections, because of their autoimmune disease as well as the immunosuppressive drugs they consume.^3^

The first purpose of this study was to evaluate the prevalence and severity of COVID-19 disease by type of collagen vascular disease. A number of drugs, such as hydroxychloroquine and anti-IL-6 such as Tocilizumab and Colchicine, were used to treat COVID-19 patients during cytokine storms stage. These drugs are regularly used in rheumatic patients.^4^

In research, we also investigated the relationship between the prevalence and severity of COVID-19 disease and types of anti-rheumatic drugs, influenza vaccine, and patient’s blood type.

## METHODS

This cross-sectional study was performed on patients referred to the rheumatology clinic of Firouzgar Hospital between August and December 2020.

Sampling method was convenience sampling method. Odds ratios were computed from 2×2 contingency tables. Inclusion criteria included all patients who were diagnosed with a specific rheumatic disease and exclusion criteria included non-rheumatic diseases such as osteoarthritis. The type of rheumatic disease and medications taken were recorded for every patient admitted to the rheumatology clinic. Every patient was asked about history of COVID-19.

Patients diagnosed with COVID-19 by PCR or ELISA and a small number due to the typical symptoms of the disease such as fever, musculoskeletal pain, sore throat, etc. Lung CT scan findings was also entered this study, patchy ground-glass change, confluent consolidation and crazy paving.

Any patient hospitalised due to COVID-19, was considered with severe disease. Patients’ blood types were recorded. At the end, they were divided into positive and negative categories in terms of influenza vaccination.

All the data were recorded and statistically analysed in SPSS 21 and measured for standard deviation (sd), frequency and p-values. A *P*-value <0.05 was considered statistically significant.

## RESULTS

Among the total of 748 patients, 574 (76.6%) cases were women, and 174 (23.3%) cases were men. The mean age of the patients was 47.46±13.56 years. In general, 60 patients (8. 0%) were diagnosed as COVID-19. The relationship between disease type and incidence of COVID-19 in other diseases is given in **Table 1**.

**Table 1.** Prevalence of COVID-19 based on the type of rheumatic disease.

**Type of Disease**	**Condition**	**Coronavirus**		**Total**	**OR (95%CI)**	**P Value**
		positive	negative			
SLE	Yes	12(20%)	110(16.2%)	122	1.298(0.668–2.523)	0.441
	No	48(80%)	571(83.8%)	619(83.5%)		
SPA	yes	3(5%)	119(17.4%)	122(16.4%)	0.249(0.077–0.810)	0.013
	No	57(95.0%)	564(82.6%)	621(83.6%)		
SSC	yes	11(18.3%)	88(12.9%)	99(13.4%)	1.513(0.758–3.020)	0.238
	No	49(81.7%)	595(87.1%)	642(86.6%)		
RA	yes	22(36.7%)	236(34.6%)	258(34.7%)	1.097(0.634–1.897)	0.742
	No	38(63.3%)	447(65.4%)	485(65.3%)		
Sjogren	yes	5(8.3%)	41(6.0%)	46(6.2%)	1.424(0.540–3.749)	0.743
	No	55(91.7%)	642(94.0%)	697(93.8%)		
GPA	yes	1(1.7%)	15(2.2%)	16(2.2%)	0.755(0.098–5.815)	0.786
	No	59(98.3%)	668(97.8%)	727(97.8%)		
Takayasu	yes	3(5%)	35(5.1%)	38(5.1%)	0.974(0.291–3.267)	0.967
	No	57(95.0%)	648(94.9%)	705(94.9%)		
Sarcoidosis	yes	3(5%)	16(2.3%)	19(2.6%)	2.194(0.621–7.754)	0.211
	No					

OR: Odds ratio; CI: Confidence interval; SLE: Systemic Lupus Erythematosus; SPA: Spondyloarthropathy; SSC: Systemic sclerosis; RA: Rheumatoid Arthritis; GPA: Granulomatosis with polyangiitis.

Out of the 60 patients diagnosed with COVID-19, 53 of them had a positive PCR, four had a positive ELISA test, and three were diagnosed based on their symptoms or CT scan results. Thirty-seven (61.6%) patients had severe COVID-19 that led to their hospitalisation. Ninety-five (12.8%) of 748 patients had been received a flu vaccine and 649 (82.8%) patients were not vaccinated. Out of 60 patients suffering from COVID-19, three (5%) had spondyloarthropathy and 2.5% of all the spondyloarthropathic patients had COVID-19, which was significant with p-value = 0.013. **This indicates a negative association between COVID disease and spondyloarthropathy.** None of the patients with COVID-19 had Behçet’s disease and p-value was considered as 0.39. **Moreover, only one patient had the APS and did not have COVID-19 disease.**

Nine (15%) cases were vaccinated against influenza, and out of 681 patients who did not suffer from coronavirus, 85 (12.5%) cases were vaccinated against influenza with a p-value of 0.54. However, there was no correlation founded between patients who received flu vaccine and COVID-19.

**Table 2** shows the prevalence of drugs used in the studied patients and their relationship with the incidence of COVID-19; as a result, no significant p-value was found. None of the patients with rheumatoid arthritis received Tocilizumab. The patients with spondyloarthropathy also did not receive Secukinumab.

**Table 2.** Prevalence of COVID-19 based on the type of drug.

**Type of Drug**	**Condition**	**Coronavirus**		**Total**	**OR (95%CI)**	**P Value**
		positive	negative			
HCQ	yes	30(50.0%)	280(41.1%)	310(41.8%)	1.436(0.846–2.436)	0.178
	no	30(50.0%)	402(58.9%)	432(58.2%)		
MTX	yes	26(43.3%)	271(39.7%)	297(40.0%)	1.163(0.682–1.981)	0.597
	no	34(56.7%)	412(60.3%)	446(60.0%)		
SSZ	yes	15(25.0%)	173(25.4%)	188(25.4%)	0.979(0.532–1.800)	0.945
	no	45(75.0%)	508(74.6%)	553(74.6%)		
Prednisolone	Upper 15mg	17(28.3%)	120(17.6%)	137(18.5%)		0.046
	Under 15mg	38(63.3%)	444(65.1%)	482(65.0%)		
	no use	5(8.3%)	118(17.3%)	123(16.6%)		
Rituximab	yes	5(8.3%)	20(2.9%)	25(3.4%)	3.009(1.087–8.327)	0.026
	no	55(91.7%)	662(97.1%)	717(96.6%)		
Altebrel (Etanercept)	yes	1(1.7%)	23(3.4%)	24(3.2%)	0.486(0.064–3.660)	0.474
	no	59(98.3%)	659(96.6%)	718(96.8%)		
Cyclophosphamide	yes	2(3.3%)	26(3.8%)	28(3.8%)	0.870(0.201–3.758)	0.825
	no	58(96.7%)	656(96.2%)	714(96.2%)		
Azaram	yes	4(6.7%)	65(9.5%)	69(9.3%)	0.678(0.238–1.930)	0.464
	no	56(93.3%)	617(90.5%)	673(90.7%)		

OR: Odds ratio; CI: Confidence interval; HCQ: Hydroxychloroquine; MTX: Methotrexate; SSZ: Sulfasalazine.

**Table 3.** The prevalence of blood groups.

		**Coronavirus**		**Total**	**P value**
		**positive**	**negative**		
Blood group	A(−)	3(%5.0%)	38(5.6%)	41(5.6%)	0.931
	A(+)	14(23.3%)	145(21.5%)	159(21.7%)	
	AB(−)	1(1.7%)	17(2.5%)	18(2.5%)	
	AB(+)	5(8.3%)	46(6.8%)	51(6.9%)	
	B(−)	1(1.7%)	20(3.0%)	21(2.9%)	
	B(+)	12(20%)	108(16%)	120(16.3%)	
	O(−)1	1(1.7%)	31(4.6%)	32(4.4%)	
	O(+)	23(38.3%)	269(39.9%)	292(39.8%)	
Total		60	674	734	
		100%	100.0%	100.0%	

Overall, 85 (11.4%) of the patients in the study had ILD (Interstitial Lung Disease) and 60 of these patients had scleroderma (70.6%). Out of 60 patients with COVID-19, 13 (21.7%) patients had ILD and 7(53.8%) of these patients had scleroderma. (**Table 2**)

## Discussion

The current study provides evidence regarding the prevalence of COVID-19 in the rheumatic and autoimmune patients referred to our centre (Rheumatology Clinic of Firouzgar Hospital). The relationship between the prevalence of COVID-19 and the type of rheumatic disease and the types of drugs and blood groups was investigated. Out of 748 patients referred to the clinic, about 8.0% developed COVID-19 in general. Based on the findings of this study, no direct relationship was observed between the type of vascular collagen disease and the prevalence of COVID-19. No significant relationship was found between influenza vaccine injection and COVID-19. The most common blood group was O positive, and no significant relationship was found between blood group and coronavirus incidence.

In our study, 17 (28.3%) of the patients who used steroids above 15 mg got COVID-19 and showed a marginal significant correlation with p-0.046, and a similar result was shown in the study of Milena Gianfrancesco et al.

In the study by Milena Gianfrancesco et al., which examined the relationship between the hospitalization rate of the rheumatic patients and COVID-19, no correlation was found between the use of rheumatic drugs and the hospitalization of the patients. However, this association was significant only with the use of more than 10 mg prednisolone per day.^5^ It may be argued that during a pandemic, patients taking steroids above 15 mg are at higher risk for viral infections, including COVID-19.

In the 2020 EULAR study of Robert BM Landewe et al., as well as our study, the prevalence of COVID-19 in rheumatic patients was not higher than general population. No significant relationship with drugs was shown in their study and they did not recommend discontinuing the drug when they were infected by COVID-19.

In spite of the study performed by Robert BM Landewe et al., this research indicated that taking steroids above 15 mg (p-value 0.046) and rituximab (p-value 0.026 OR 3.009) was associated with a higher prevalence of COVID-19.

In their study, Silva et al. also illustrated that ICU hospitalization and need for ventilation was higher in the rheumatic patients than the general public. However, the prevalence of the disease and their hospitalization rates were similar to the general population.^7^

Obviously, comparing outcome of this research with above results reflects a huge difference. There are several reasons that justify these discrepancies:

- The mean age of patients in this study was much higher (62.5 ± 15.1) and at this age there is a higher risk of hospitalisation and severe disease from COVID-19
- 63% of their patients had active rheumatic disease at the time of COVID-19 and therefore they had to use higher doses of immunosuppressive drugs, which was lower in our patients
- 37% of their patients used biological drugs.

A study done by Ennio Giulio Favalli et al. in Italy in 2020 on 123 people with collagen vascular disease also showed that the prevalence of COVID-19 was not higher in these patients, so they did not need to discontinue their medication.^8^

This was very similar to the result achieved in this research.

Another study done by Fernando Montero et al. in 2020 showed that the patients with pulmonary involvement and high dose of steroids had a slight increase in hospitalization rate compared to the general population.^9^

These conclusions were also in line with the results of this study.

In a study conducted by Clodoveo Ferri et al. in 2020 on 1641 patients in three centres, the prevalence of COVID-19 was higher in autoimmune patients. The discrepancy could be due to the fact that patients’ information about COVID-19 was collected by telephone calls. Number of patients did not have positive PCR for COVID-19.

However, their study showed that, the severity of the disease was higher in those with ILD, similar to the outcome of our research.^10^

In summary, this study showed that the prevalence of COVID-19 in rheumatic disease were not higher than those of the general population. In addition, there was no significant relationship among the type of disease, drugs taken, and blood type of the patients and the prevalence and severity of COVID-19 in these rheumatic patients.

## References

[1] Arkadiusz S Karolina O Zaneta K Bozena L. Cytokine Storms in the Course of COVID-19 and Haemophagocytic Lymphohistiocytosis in Pregnant and Postpartum Women. Biomolecules 2021;11(8):1202. 34439868 10.3390/biom11081202 PMC8391528

[2] Zhou P Yang X-L Wang X-G Hu B Zhang L Zhang W A pneumonia outbreak associated with a new coronavirus of probable bat origin. Nature 2020;579:270–3. 32015507 10.1038/s41586-020-2012-7 PMC7095418

[3] Favalli EG Biggioggero M Meroni PL. Methotrexate for the treatment of rheumatoid arthritis in the biologic era: still an “anchor” drug? Autoimmun Rev 2014;13:1102–8. 25172238 10.1016/j.autrev.2014.08.026

[4] Tisoncik JR Korth MJ Simmons CP Farrar J Martin TR Katze MG. Into the eye of the cytokine storm. Microbiol Mol Biology Rev Mmbr 2012;76:16–32. 10.1128/MMBR.05015-11 PMC3294426 22390970

[5] Gianfrancesco M Hyrich KL Al-Adely S Carmona L Danila MI Gossec L Characteristics associated with hospitalisation for COVID-19 in people with rheumatic disease: data from the COVID-19 Global Rheumatology Alliance physician-reported registry. Ann Rheum Dis 2020 Jul; 79(7):859–66. 32471903 10.1136/annrheumdis-2020-217871 PMC7299648

[6] Landewé RB Machado PM Kroon F Bijlsma HW Burmester GR Carmona L EULAR provisional recommendations for the management of rheumatic and musculoskeletal diseases in the context of SARS-CoV-2. Ann Rheum Dis. 2020 Jul; 79(7):851–858. 32503854 10.1136/annrheumdis-2020-217877

[7] D’Silva KM Serling-Boyd N Wallwork R Hsu T Fu X Gravallese EM Clinical characteristics and outcomes of patients with coronavirus disease 2019 (COVID-19) and rheumatic disease: a comparative cohort study from a US ’hot spot’. Ann Rheum Dis 2020 Sep;79(9):1156–62. 32457048 10.1136/annrheumdis-2020-217888 PMC7456555

[8] Favalli EG Agape E Caporali R. Incidence and Clinical Course of COVID-19 in Patients with Connective Tissue Diseases: A Descriptive Observational Analysis. J Rheumatol 2020 Aug 1;47(8):1296. 32335513 10.3899/jrheum.200507

[9] Montero F Martínez-Barrio J Serrano-Benavente B González T Rivera J Molina Collada J Coronavirus disease 2019 (COVID 19) in autoimmune and infammatory conditions: clinical characteristics of poor outcomes. Rheumatology International 2020;40:1593–8. 32794113 10.1007/s00296-020-04676-4 PMC7425254

[10] Ferri C Giuggioli D Raimondo V L’Andolina M Tavoni A Cecchetti R COVID-19 and rheumatic autoimmune systemic diseases: report of a large Italian patients series. Clin Rheumatol 2020;39:3195–204. 32852623 10.1007/s10067-020-05334-7 PMC7450255

